# Flash visual evoked potentials (FVEP) in various stimulation conditions

**DOI:** 10.1007/s10633-018-9663-9

**Published:** 2018-11-23

**Authors:** Dorota Pojda-Wilczek, Wojciech Maruszczyk, Sebastian Sirek

**Affiliations:** Ophthalmology Clinic and Department of Ophthalmology, University Clinical Centre, School of Medicine in Katowice, Medical University of Silesia in Katowice, Ceglana 35, 40-514 Katowice, Poland

**Keywords:** Flash visual evoked potentials, Ganzfeld, Mini Ganzfeld, Flash Goggles

## Abstract

**Aim:**

To compare flash visual evoked potentials (FVEP) elicited using a Ganzfeld bowl (G), Mini Ganzfeld (MG) and Flash Goggles (GG) with eyes open and closed.

**Patients and method:**

The study group comprised 17 volunteers with mean age of 30 years; all of them were examined with the Roland Consult electrophysiological diagnostic system. Active electrodes were placed at O_1_ and O_2_. With the G and MG stimulators, the flash generated by white-light-emitting diodes (LEDs) presented standard flash of 3 cd s m^−2^. The GG used red LED flash of 3 cd s m^−2^. Stimulus frequency of 1.0 Hz, low-pass filter of 1.0 Hz and high-pass filters of 100 Hz (G); 50 Hz (MG); 30 Hz (GG) were used. P2 amplitude and latency were compared by the means of the Wilcoxon matched-pairs signed-rank test.

**Results:**

After right eye stimulation (from O_1_; *n* = 17), the mean amplitudes of P2, elicited with the G, MG and GG, were 13, 7 and 10 µV, respectively. The respective latencies were 129, 114 and 110 ms. Hence, the difference between the results obtained with these stimulators was statistically significant (*p* < 0.05). The mean P2 amplitudes, acquired by the means of the G, MG and GG, were 13 µV, 7 µV and 10 µV for open eyes, and 11 µV, 8 µV and 8 µV for closed eyes. The respective latencies were 129, 114 and 110 ms for eyes open, and 127, 125 and 121 ms for eyes closed. These results of the MG (latency only) and GG (latency and amplitude) stimulation differed significantly (*p* < 0.05).

**Conclusion:**

The amplitudes and latencies of the FVEP P2 elicited with different stimulators are not suitable for comparison. Closing the eye during the examination had a significant effect on the components of FVEP waveform elicited with the Flash Goggle and on the latency of P2 elicited with the MG.

## Introduction

Flash visual evoked potentials (FVEP) have been used in ophthalmology and neurology since 1960s. Nowadays, the flash stimulus may not be considered to be the first choice in obtaining VEP, since pattern visual evoked potentials (PVEP) are much more reproducible and consistent among patients. However, PVEP require stable fixation, good cooperation and correction of refractive errors. Thus, this type of stimulation is sometimes difficult to perform in babies and young children, especially in the presence of a developmental disability or severe vision impairment. In the presence of developmental disability compliance and understanding are limited and therefore standard vision assessment techniques are not possible regardless of age. Therefore, for many patients flash stimulation may be the optimum tool for visual evoked potential assessment.

Apart from the standard Ganzfeld stimulator, other stimulators, such as the Mini Ganzfeld, Flash Baby, Flash Goggles, are used, especially for the examination of infants and children. Also a variety of different devices in use to assess FVEP in different attentional states of the patient are used. Intraoperative monitoring using VEP has been described. Benedičič and Bošnjak [1] used the Flash Goggles, while Luo et al. the light-stimulating device [2] for intraoperative assessment of the integrity of intracranial visual pathways. The conditions of stimulation vary from one laboratory to another one. Chayasirisobhon et al. [3] used LED goggles to assess awake neonates, while Klebermass-Scherehof et al. [4] and Feng et al. [5] examined infants during sleep. Jethani and Jethani used standard handheld stimulation and examination under sedation [6], and Shepherd et al. examined infants in different states of sleep using stroboscopic flash [7].

When interpreting FVEP, the type of device needs to be considered and also the use of anesthesia, the state of patient alertness (asleep, alert) and whether eyes are open or closed, e.g., Tartaglione, Bandini et al. 1995 “Eye Closure affects flash VEP latency in dementia” [8]. This study demonstrated particular latency delays of the P2 (eyes closed 3 ms delayed compared with eyes open) versus other components with the delay being significant in those with dementia versus control. All these factors need to be considered in the interpretation of flash VEP.

There is a lack of studies juxtaposing the results of examinations using various stimulators, and only some studies concentrate on the differences between examinations with open and closed eyes [9]. The aim of this study is to clarify whether results from FVEP stimulators differ and the effect of examination in the waking (open eye) versus closed eye state.

## Aim

The aim of the study was to compare the flash visual evoked potentials (FVEP) elicited using the Ganzfeld bowl, Mini Ganzfeld and Flash Goggles with eyes open and closed.

## Patients and methods

Seventeen volunteers (34 eyes), including 11 females, aged 23–56 (mean age: 30 years) were examined by the means of the Reti-port (Roland Consult, Germany) electrophysiological equipment. The examination was performed in a dimly illuminated room. The flash visual evoked potentials (FVEP) were examined using the following stimulators: the Ganzfeld bowl, Mini Ganzfeld and Flash Goggles. Each stimulator was used to conduct the following examinations: right eye open, left eye open and then eyes closed starting from the right one.

During the examination using the Ganzfeld bowl and the Mini Ganzfeld, the fellow eye was completely patched by a black, carton obturator with a gauze tampon on the closed eyelid. In the case of the Flash Goggles examination, the eyes were not covered.

The pupils were not dilated. According to the International 10/20 system, two active gold cup electrodes were placed in O_1_ and O_2_ points in relation to the electrode located in Fz. A ground electrode (gold cup) was located on the forehead. The examination conditions were as follows: stimulus frequency 1.0 Hz, cycle time 1 s, background light off, automatic artifact rejection of signals exceeding ± 50–100 µV in amplitude, low-pass filter 1.0 Hz and high-pass filters: 100 Hz (Ganzfeld bowl) or 50 Hz (Mini Ganzfeld) or 30 Hz (Flash Goggles), average of 100 sweeps. With the Ganzfeld bowl and Mini Ganzfeld stimulators, the flash generated by white-light-emitting diodes (LEDs) presented standard flash of 3 cd s m^−2^. The Flash Goggles used red LED flash of 3 cd s m^−2^. Flash duration in Ganzfeld bowl was 0.91 s; in Mini Ganzfeld and Flash Goggles, it was 1 s. Amplifier V2.0 with original settings for each stimulator (done by Roland Consult) was used.

Measurements of the P2 amplitude was taken from the preceding N2 negative peak to positive P2 peak at around 120 ms (from 100 to 140 ms). The P2 wave amplitude after initial Ganzfeld stimulation with eye open was higher than 5 µV at least from one of two electrodes (O_1_ and O_2_). The P2 wave was identified as the largest peak between 100 and 140 ms after stimulation.

Using the Wilcoxon signed-rank test, the amplitude and the latency of the P2 wave, obtained from electrode locations O_1_ and O_2_ separately after right eye (RE) and left eye (LE) stimulation (*n* = 17), were compared. The first comparison concerned the results acquired with each of the three stimulators and the second the results acquired with open and closed eyes.

## Results

**Fig. 1:**
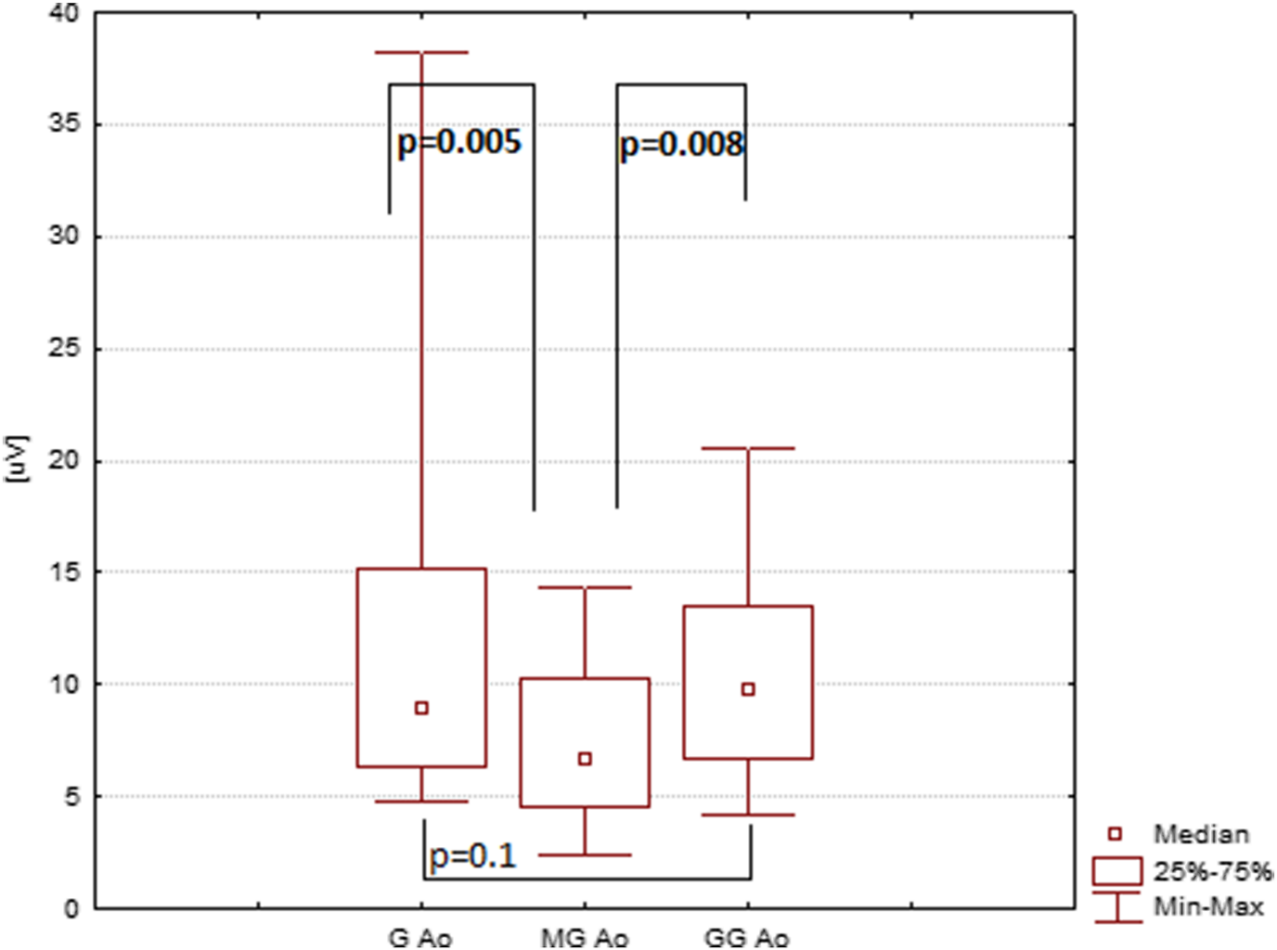
P2 amplitude in examination with the Ganzfeld bowl (G), Mini Ganzfeld (MG) and Flash Goggles (GG). Eyes open, right eye, electrode at O_1_

**Fig. 2:**
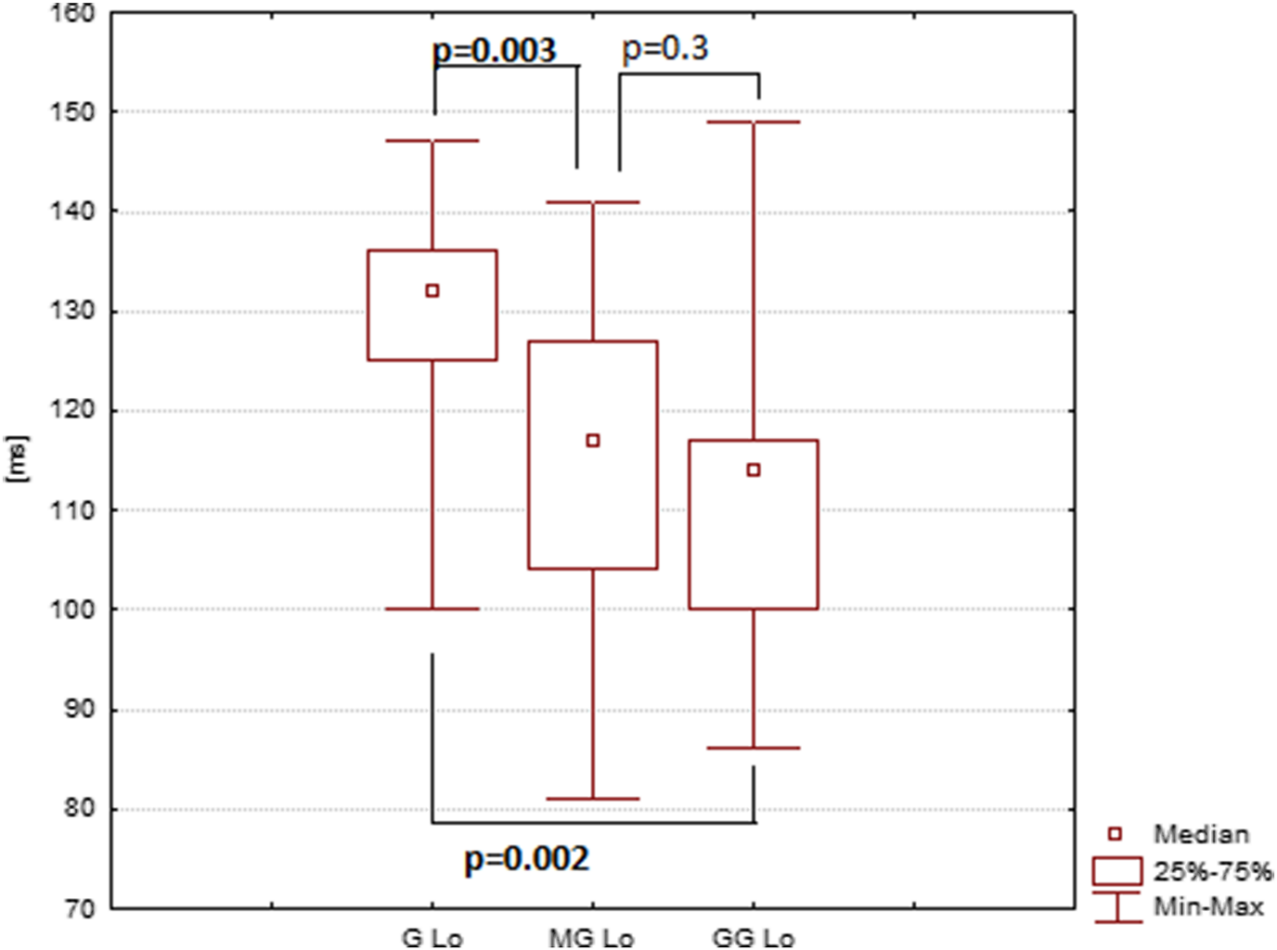
P2 latency in examination with the Ganzfeld bowl (G), Mini Ganzfeld (MG) and Flash Goggles (GG). Eyes open, right eye, electrode at O_1_

The mean P2 amplitude (latency) acquired with the Ganzfeld bowl stimulation with open eyes (right eye, electrode at O_1_) amounted to 13 µV (129 ms), that acquired with the Mini Ganzfeld stimulation to 7 µV (114 ms), and that acquired with the Flash Goggles to 10 µV (110 ms) (Tables 1, 2; Figs. 1, 2). Statistically, the differences of P2 amplitude between Ganzfeld bowl and Mini Ganzfeld and between Mini Ganzfeld and Flash Goggles were significant (*p* = 0.005 and *p* = 0.008, respectively). The difference between Ganzfeld bowl and Flash Goggles was not significant (*p* = 0.1). The differences of P2 latency between Ganzfeld bowl and Mini Ganzfeld and between Ganzfeld bowl and Flash Goggles were significant (*p* = 0.003 and *p* = 0.002, respectively). The difference between Mini Ganzfeld and Flash Goggles was not significant (*p* = 0.3).

**Table 1:** P2 amplitude in examinations with the Ganzfeld bowl, Mini Ganzfeld and Flash Goggles with eyes open (*n* = 17)

Amplitude P2 [μV] RE O_1_	Average	Median	Min.	Max.	SE	Amplitude P2 [μV] LE O_1_	Average	Median	Min.	Max.	SE
Ganzfeld	13.5	8.9	4.8	38.2	2.44	Ganzfeld	12.4	12.4	5.8	20.8	1.06
Mini Ganzfeld	7.3	6.6	2.4	14.3	0.89	Mini Ganzfeld	7.2	6.7	1.3	17.8	0.98
Flash Goggles	10.2	9.8	4.1	20.6	1.08	Flash Goggles	9.1	8.3	3.7	15.4	0.83

Amplitude P2 [μV] RE O_2_	Average	Median	Min.	Max.	SE	Amplitude P2 [μV] LE O_2_	Average	Median	Min.	Max.	SE
Ganzfeld	14.9	12.8	2.3	40.3	2.49	Ganzfeld	13.9	12.8	4.9	27.7	1.43
Mini Ganzfeld	8.1	8.3	0.9	12.7	0.71	Mini Ganzfeld	8.8	9.1	0.4	18	1.02
Flash Goggles	11.5	10.3	5.7	19.8	0.95	Flash Goggles	10.9	10.5	4.6	21.9	1.16

**Table 2:** P2 latency in examinations with the Ganzfeld bowl, Mini Ganzfeld and Flash Goggles with eyes open (*n* = 17)

Latency P2 [ms] RE O_1_	Average	Median	Min.	Max.	SE	Latency P2 [ms] LE O_1_	Average	Median	Min.	Max.	SE
Ganzfeld	129	132	100	147	3.03	Ganzfeld	126	126	104	148	3.37
Mini Ganzfeld	114	117	81	141	4.02	Mini Ganzfeld	122	127	81	142	3.68
Flash Goggles	110	114	86	149	3.96	Flash Goggles	112	113	88	149	3.77

Latency P2 [ms] RE O_2_	Average	Median	Min.	Max.	SE	Latency P2 [ms] LE O_2_	Average	Median	Min.	Max.	SE
Ganzfeld	129	132	100	147	3.06	Ganzfeld	125	123	103	148	3.56
Mini Ganzfeld	119	124	79	142	3.89	Mini Ganzfeld	120	127	81	136	3.71
Flash Goggles	112	115	81	146	4.3	Flash Goggles	112	112	91	144	3.29

**Fig. 3:**
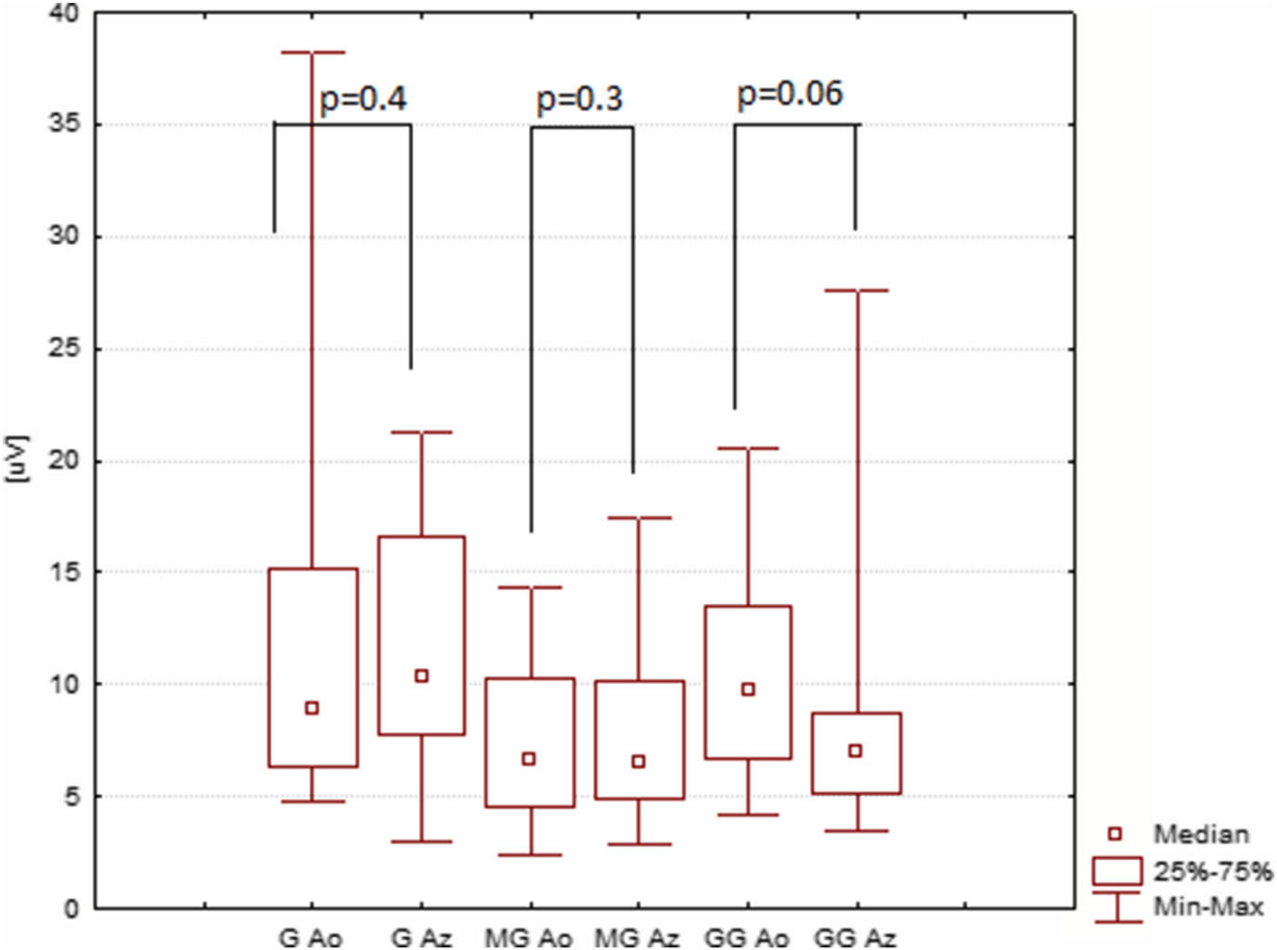
The differences between P2 amplitudes in examinations with eyes open (Ao) and closed (Az). Ganzfeld bowl (G), Mini Ganzfeld (MG), Flash Goggles (GG); right eye, electrode at O_1_

**Fig. 4:**
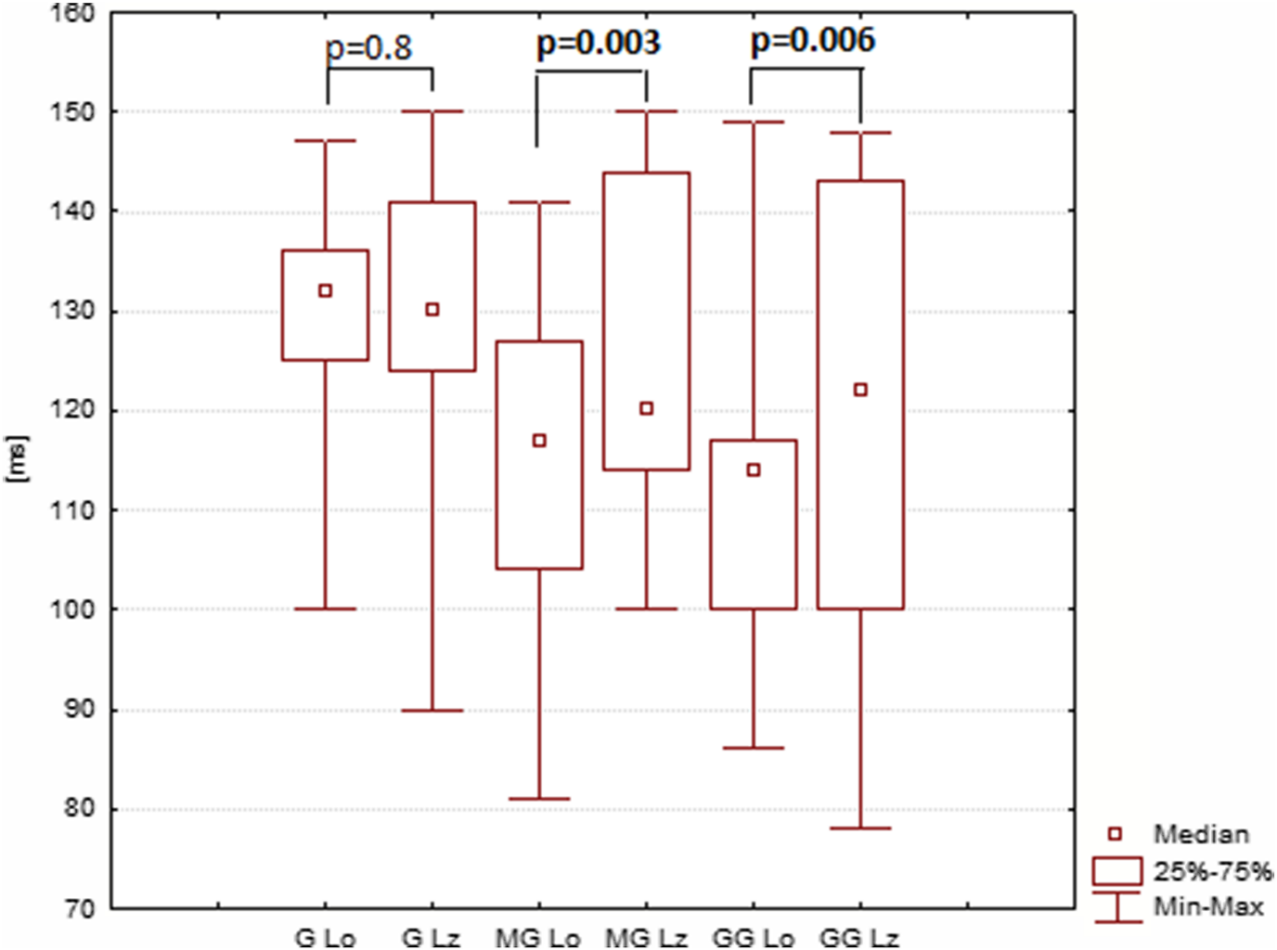
The differences between P2 latencies in examinations with eyes open (Lo) and closed (Lz). Ganzfeld bowl (G), Mini Ganzfeld (MG), Flash Goggles (GG); right eye, electrode at O_1_

**Fig. 5:**
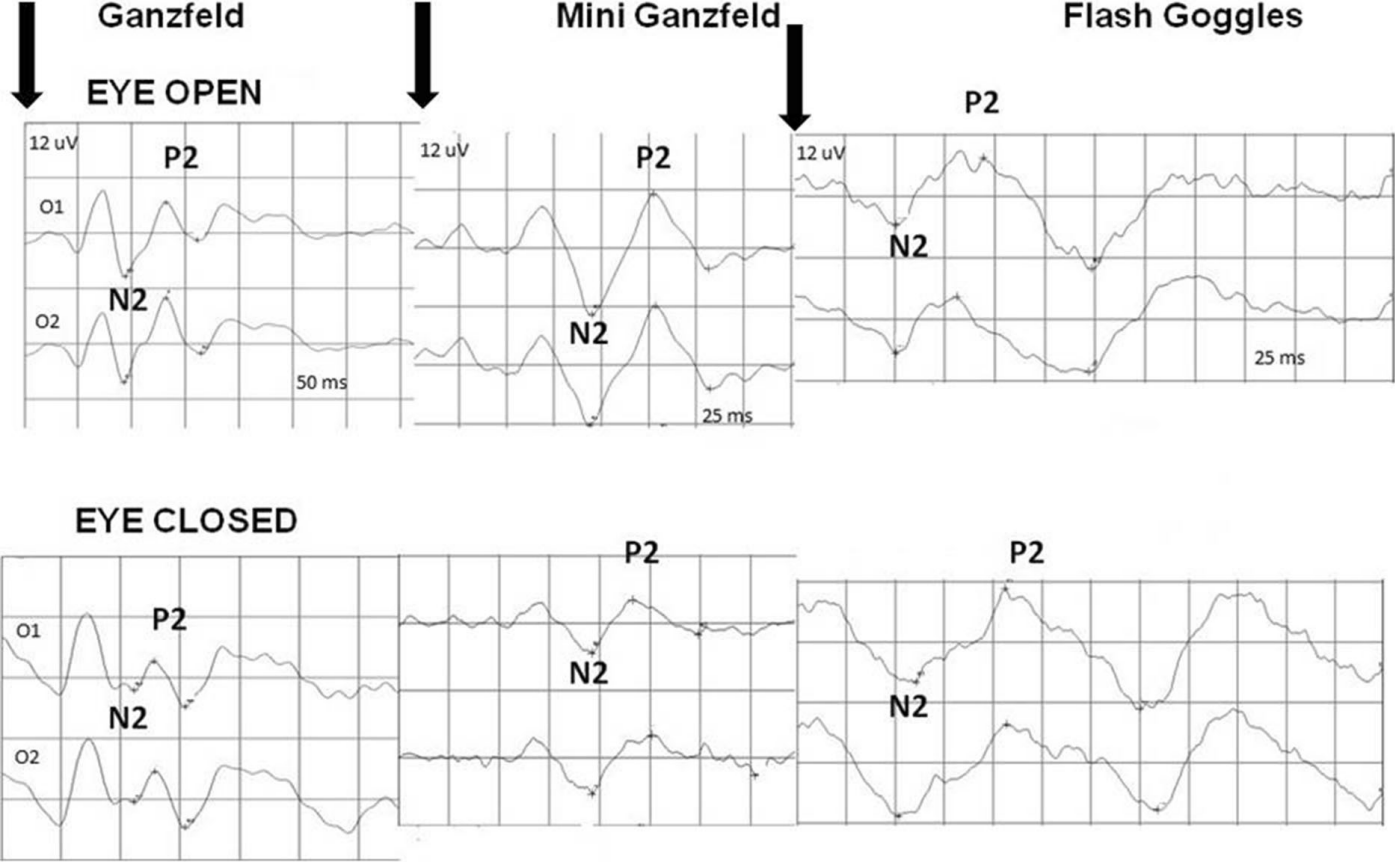
An example of examination with various types of stimulators and the same eye open and closed. The arrows indicate the beginning of stimulation

The mean P2 amplitude (latency) acquired with the Ganzfeld bowl stimulation with eyes closed amounted to 11 µV (127 ms), that acquired with the Mini Ganzfeld stimulation to 8 µV (125 ms), and that acquired with the Flash Goggles to 8 µV (121 ms) (Tables 3, 4). The results for open and closed eyes were, from a statistical point of view, significantly different in the case of both the Mini Ganzfeld (P2 latency: *p* = 0.003; P2 amplitude: *p* = 0.3) and the Flash Goggles (P2 latency: *p* = 0.006; P2 amplitude: *p* = 0.06) stimulators (Figs. 3, 4, 5). No significant differences between the results for open and closed eyes acquired with the Ganzfeld bowl were found (P2 latency: *p* = 0.8; P2 amplitude *p* = 0.4).

**Table 3:** P2 amplitude in examinations with the Ganzfeld bowl, Mini Ganzfeld and Flash Goggles with eyes closed (*n* = 17)

Amplitude P2 [μV] RE O_1_	Average	Median	Min.	Max.	SE	Amplitude P2 [μV] LE O_1_	Average	Median	Min.	Max.	SE
Ganzfeld	11.6	10.4	2.9	21.3	1.34	Ganzfeld	11.3	11.3	2.26	21.4	1.32
Mini Ganzfeld	8.2	6.5	2.8	17.5	1.04	Mini Ganzfeld	9.4	9.2	1.3	18.2	1.11
Flash Goggles	8.4	6.9	3.5	27.6	1.38	Flash Goggles	7.1	5.9	2.3	21.1	1.13

Amplitude P2 [μV] RE O_2_	Average	Median	Min.	Max.	SE	Amplitude P2 [μV] LE O_2_	Average	Median	Min.	Max.	SE
Ganzfeld	11.6	11.5	2.7	19.9	1.5	Ganzfeld	13.3	12.2	0.64	24.9	1.87
Mini Ganzfeld	9.8	11	1.8	19.2	1.17	Mini Ganzfeld	10.6	10.7	0.72	22.7	1.42
Flash Goggles	8.1	7.7	1.7	25.4	1.39	Flash Goggles	8.2	6	1.5	22.8	1.31

**Table 4:** P2 latency in examinations with the Ganzfeld bowl, Mini Ganzfeld and Flash Goggles with eyes closed (*n* = 17)

Latency P2 [ms] RE O_1_	Average	Median	Min.	Max.	SE	Latency P2 [ms] LE O_1_	Average	Median	Min.	Max.	SE
Ganzfeld	127	130	90	150	4.1	Ganzfeld	130	139	100	155	4.23
Mini Ganzfeld	125	120	100	150	4.1	Mini Ganzfeld	130	137	105	150	4.26
Flash Goggles	121	122	78	148	5.65	Flash Goggles	119	120	92	156	5.52

Latency P2 [ms] RE O_2_	Average	Median	Min.	Max.	SE	Latency P2 [ms] LE O_2_	Average	Median	Min.	Max.	SE
Ganzfeld	129	132	90	151	4.24	Ganzfeld	128	131	100	145	3.66
Mini Ganzfeld	124	124	101	149	3.53	Mini Ganzfeld	131	139	106	151	4.11
Flash Goggles	121	125	83	146	5.15	Flash Goggles	118	120	88	156	5.11

## Discussion

The increasing number of patients with hypoxic brain damage can contribute to the fact that the FVEP will be useful as a prognostic value not only for children, but also for adult patients [10]. The conditions of the examinations have to be often adjusted to the patient; for example, the examination can be conducted in a supine position. When the improved health condition and cooperation with the examined patient make it possible to conduct a control examination in the standard conditions, it is important to bear in mind that even the same flash frequency, even though obtained by the means of a different type of stimulator, will trigger a response of a different value of latency and amplitude.

In the examination using the Mini Ganzfeld and Flash Goggles, the P2 amplitudes were lower than after the stimulation with the Ganzfeld bowl. P2 latency was longest after the Ganzfeld bowl stimulation and shortest after the Flash Goggles. The differences between particular stimuli stem mainly from the difference in the settings of amplifiers and stimulus wavelength characteristics.

It is easier to explain the differences between responses obtained with open and closed eyes. Decreased flash luminance, as a result of closed eyelids, was the reason for the extension of the wave latency. Such was also the conclusion following from the study conducted by Subramanian et al. [11]. At the same time, the researchers proved a higher FVEP reproducibility and result stabilization in the consecutive examinations with closed eyes. Therefore, they suggest conducting the FVEP with closed eyes.

In the present study after closing eyes, VEPs in the majority of cases became more regular, almost sinusoidal with not so sharp and clearly mark peaks. It cannot be ruled out that the decrease in the quality of the flash, in the case of closed eyes, caused a weaker response to stimulation, with a simultaneously recorded higher basic brain activity. Halliday recorded sinusoidal discharge after flash stimulation and described its potential connection with the resting rhythm alpha EEG (electroencephalography) [12]. The alpha rhythm is recorded, among others, in coma [13]. The lack of fixation in the case of closed eyes and reduced alertness could have caused the visual response to be significantly modified by the increase in a different activity of the brain. Opening eyes probably causes this activity to be suppressed by a dominant activity of the visual system. A lower P2 amplitude after stimulation through eyelid should be expected because of lower intensity of flash. On the other hand, superimposition of signals evoked by flash and other brain rhythms in some conditions may be an explanation of higher P2 amplitude when eyes are closed.

A different response after the Flash Goggles stimulation can also result from the application of a dozen or so many red light diodes in this stimulator. In the clinical practice, it is crucial to standardize the means of stimulation in order to enable the juxtaposition of results obtained from a patient in different age and clinical condition. It is particularly important in, for example, children and adults with hydrocephalus, craniostenosis or brain tumors, examined repeatedly in connection with the possibility of occurrence of intracranial hypertension.

Studies by Sjöstrom et al. [14] and Desch [15] constitute examples of applying the VEP to monitor the intracranial pressure. Applying the Ganzfeld bowl stimulation from the very beginning seems to be the best choice, even in young children. The Ganzfeld bowl examination gives the child the biggest freedom, contact with the stimulator is minimal, and the examination does not require sedation.

The results of this study should be of interest within the context of multicenter clinical trials and in clinical practice when a given patient’s condition is evaluated on the basis of the FVEP results obtained at different times and using different methods.

The current study had numerous limitations: The examination lasted about 30 min per patient, and stimulations were not repeated. Samples are small, and in some cases, the differences did not reach statistical significance.

## Conclusion

The amplitudes and latencies of the FVEP P2 elicited with different stimulators are not suitable for comparison. Closing the eye during the examination had a significant effect on the components of FVEP waveform elicited with the Flash Goggle and on the latency of P2 elicited with the Mini Ganzfeld.
